# Higher circulating Trimethylamine N-oxide levels are associated with worse severity and prognosis in pulmonary hypertension: a cohort study

**DOI:** 10.1186/s12931-022-02282-5

**Published:** 2022-12-14

**Authors:** Yicheng Yang, Beilan Yang, Xin Li, Lin Xue, Bingyang Liu, Yanru Liang, Zhihui Zhao, Qin Luo, Zhihong Liu, Qixian Zeng, Changming Xiong

**Affiliations:** Center of Respiratory and Pulmonary Vascular Disease, State Key Laboratory of Cardiovascular Disease, Fuwai Hospital, National Center for Cardiovascular Disease, Chinese Academy of Medical Sciences and Peking Union Medical College, No. 167 North Lishi Road, Xicheng District, Beijing, 100037 China

**Keywords:** Trimethylamine N-oxide, Biomarker, Pulmonary hypertension, Prognosis, Cardiovascular diseases

## Abstract

**Background:**

Trimethylamine N-oxide (TMAO), the gut microbiota-dependent metabolite, is a potential biomarker in several cardiovascular diseases. However, no study has investigated its value in pulmonary hypertension (PH). Therefore, this study aimed to explore the association between plasma TMAO levels and prognosis in patients with PH.

**Methods:**

Inpatients with idiopathic/heritable pulmonary arterial hypertension (IPAH/HPAH), PAH associated with congenital heart disease (CHD-PAH), and chronic thromboembolic pulmonary hypertension (CTEPH) at Fuwai Hospital were enrolled after excluding those with relative comorbidities. The endpoint was defined as a composite outcome including death, rehospitalisation due to heart failure, and at least 15% decreased 6-min walk distance from the baseline. Fasting blood samples were collected to measure plasma levels of TMAO and other clinical indicators. The associations between TMAO levels with disease severity and patients’ prognosis were investigated.

**Results:**

In total, 163 patients with PH were included, with a mean follow-up duration of 1.3 years. After adjusting for confounding factors, elevated TMAO levels were still associated with severe disease conditions. TMAO levels dynamically decreased in stable and improved patients after treatment [ΔTMAO = − 0.2 (− 1.6, 0.7) μmol/L, *P* = 0.006]. Moreover, high plasma TMAO levels predicted a poor prognosis in the PH cohort (*P* < 0.001), and the association remained significant after adjusting the confounders, including treatment, risk stratification, and PH subtypes.

**Conclusion:**

Elevated plasma TMAO levels were associated with severe disease conditions and poor prognosis in patients with PH, indicating its potential biomarker role in PH.

**Supplementary Information:**

The online version contains supplementary material available at 10.1186/s12931-022-02282-5.

## Introduction

Pulmonary hypertension (PH), generally defined as an increase in mean pulmonary arterial pressure (mPAP) ≥ 25 mmHg [1], is a rare but devastating disease for which treatment remains challenging. End-stage PH is often characterised by severe asthenia and significantly reduced heart function, eventually leading to death. Despite the shared characteristic of elevated pulmonary arterial pressure, PH is a clinically heterogeneous disease that can be divided into several groups according to the 2015 European Society of Cardiology guidelines [1]. These groups can differ greatly in terms of aetiology, pathogenesis, and clinical findings, indicating the complexity of clinical management. Therefore, effective indicators of PH severity and patient prognosis are vital for disease improvement. The search for pathogenic mechanisms and sensitive plasma biomarkers in PH remains important to date.

Trimethylamine N-oxide (TMAO) is a common metabolite in the blood mainly derived from the microbiota-dependent metabolic processing of dietary precursors in the intestine [2]. Accumulating evidence from recent studies suggests a correlation between TMAO and several diseases, including metabolic disorders, hypertension, stroke, and atherosclerosis [3–6]. Notably, such studies have indicated that TMAO is involved in the pathogenesis of many cardiovascular diseases, especially heart failure, in which it is an independent predictor of all-cause mortality [7].

However, the value of TMAO in patients with PH remains unknown. Based on the above findings, this study explored the associations between TMAO levels and PH, including idiopathic/heritable pulmonary arterial hypertension (IPAH/HPAH), pulmonary arterial hypertension associated with congenital heart disease (CHD-PAH), and chronic thromboembolic PH (CTEPH). We hypothesised that elevated plasma TMAO levels were associated with disease severity and poor prognosis in patients with PH of different aetiologies.

## Materials and methods

This clinical study was approved by Fuwai Hospital (Beijing, China) and adhered to the Declaration of Helsinki. Written informed consent was obtained from all patients.

### Study design

#### Patient selection and clinical data collection

PH was diagnosed as mPAP of  ≥ 25 mmHg via right heart catheterisation. Inpatients with IPAH/HPAH, CHD-PAH, or CTEPH treated in the Center of Pulmonary Vascular Ward of Fuwai Hospital from May 2019 to February 2020 were considered for this study. To address potential bias, we excluded PH patients: (a) with immune disease, active infection, acute coronary syndrome, malignancy, left heart failure, and diabetes; (b) who had ever-received targeted drugs; (c) who had ever-received pulmonary endarterectomy (PEA) and/or balloon pulmonary angioplasty (BPA); and (e) those lost to follow-up.

Data related to demographic characteristics, World Health Organization Functional Class (WHO-FC), biochemical indicators at fasting, TMAO levels at fasting, echocardiography findings, and haemodynamic variables were collected.

#### Follow-up and study endpoint

PH patients in this study received at least two hospitalisations. The circulating TMAO levels were explored during both hospital visits in Fuwai Hospital (with a mean of 5.6 months). After the second discharge, patients were followed up by clinical visits or telephone calls. The endpoint of this study was defined as a composite outcome of events including death, rehospitalisation due to heart failure, and at least 15% decreased 6-min walk distance (6MWD) from the baseline [8]. Follow-up duration was defined as the period from the first TMAO examination to the occurrence of outcomes or the end of follow-up.

#### Quantification of TMAO

Fasting blood samples were collected and centrifuged for 10 min at 3000 rpm to obtain supernatants for TMAO detection. Plasma (20 μL) was aliquoted into a tube and mixed with 80 μL of 10-μM internal standard composed of d9-metabolites in methanol. After vortexing for 1 min, the proteins in the samples were precipitated, and the supernatant was retrieved via centrifugation at 20,000 ×*g* at 4 °C for 10 min. To obtain the precise concentration of the analytes, 20 μL of various concentration standards (0–100 μM) processed in parallel were used to obtain an acceptable standard curve until the coefficient of determination (R2) reached 0.99. At a flow rate of 0.5 mL/min, the supernatants (70 μL) were injected onto a silica column (2.0 × 150 mm; Luna 5u Silica 100A; cat. no. 00F-4274-B0; Phenomenex, Torrance, CA, USA) using an LC-20AD Shimadazu pump system and a SIL-20AXR autosampler interfaced with an API 5500Q-TRAP mass spectrometer (AB SCIEX, Framingham, MA, USA).

Solvent A (0.1% propanoic acid in water) was mixed with solvent B (0.1% acetic acid in methanol) at different ratios beginning from 2% B linearly to 95% B over 5 min, held for 1 min, and then back to 2% B to resolve the analytes by generating a discontinuous gradient. Analytes were monitored using electrospray ionisation in the positive-ion mode with multiple reaction monitoring of the precursor and characteristic product-ion transitions of TMAO at m/z 76 → 58 and d9-TMAO at m/z 85 → 66. Three quality control samples with different TMAO concentrations were detected for every 20 samples [9].

### Statistical analysis

Continuous variables were presented as mean ± standard deviation or median and interquartile ranges based on different data distributions; categorical variables were presented as frequencies with percentages. Restricted cubic spline was used to demonstrate the association between plasma TMAO levels and endpoints preliminarily. The surv_cutpoint function was used to explore the cut-off values [10] of TMAO for dividing patients into high and low TMAO groups. The differences between groups were explored using Student’s t-test or the Wilcoxon rank-sum test for continuous variables and the chi-square test for categorical variables. Paired-samples t-tests were used to detect changes in TMAO before and after treatment. The correlations between TMAO and clinical markers of PH severity were determined using Spearman’s correlation analyses (two-tailed), univariate or multivariate logistic regression, and linear regression analysis. Kaplan–Meier analysis and Cox proportional hazards regression were used for prognostic analyses in patients with PH. A two-sided *P* value of < 0.05 was considered statistically significant. R 2.8.0 (Vienna, Austria), SPSS (version 23; IBM Corp, 2015; Armonk, NY, USA), and GraphPad (GraphPad Software, Inc., San Diego, CA, USA) were used in this study.

## Results

### Study population at baseline

**Fig. 1:**
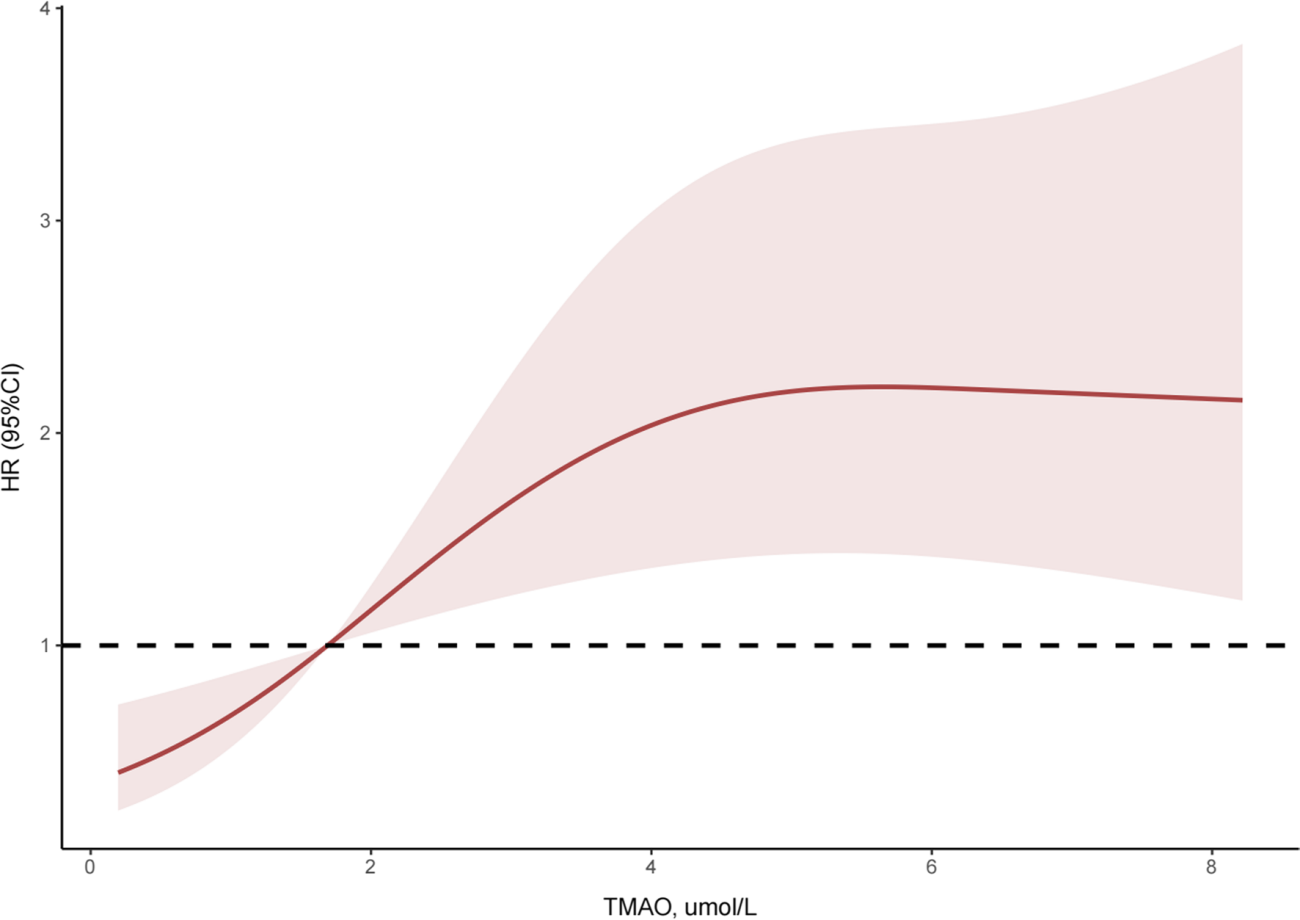
Restricted cubic spline of TMAO levels in relation to hazard ratio for the risk of clinical endpoint (n = 163). Dark red line with 95% confidence interval shaded in light red. *HR* hazard ratio; *CI* confidence interval; *TMAO* trimethylamine-N-oxide

From May 2019 to February 2020, 347 inpatients were diagnosed with IPAH/HPAH, CHD-PAH, or CTEPH. Among them, 173 individuals were excluded due to comorbidities and/or having received specific treatment, while 11 patients were lost to follow-up. Finally, 163 patients with PH, including 36 with IPAH/HPAH, 76 with CHD-PAH, and 51 with CTEPH, were included in this study. In addition, 44 CTEPH patients received PEA or BPA treatment after enrolment. During a mean of 1.3 ± 0.9 years of follow-up, 112 patients survived without clinical events, 45 were rehospitalised due to heart failure or decreased exercise capacity, and 6 died. A restricted cubic spline was used to intuitively display the association between plasma TMAO levels and composite endpoints (Fig. 1). The optimal cut-off explored by the surv_cutpoint function was 1.69 μmol/L (Additional file 1: Fig. S1), and patients were then divided into two cohorts. Mean TMAO levels in the total cohort, high-TMAO group, and low-TMAO group were 2.0 (1.0, 3.9) μmol/L, 3.7 (2.4, 5.6) μmol/L, and 0.8 (0.4, 1.3) μmol/L, respectively (Table 1). Individuals stratified into the high-TMAO group had worse WHO-FC, higher N-terminal pro-brain natriuretic peptide (NT-proBNP) levels and pulmonary vascular resistance (PVR), shorter 6MWD, larger right ventricular diameter (RVD), and lower tricuspid annular plane systolic excursion (TAPSE) and cardiac index (CI) than those in the low-TMAO group, which indicated more severe disease conditions in PH.

**Table 1:** Baseline characteristics of patients with PH stratified by TMAO levels

Variables	Total PH patients N = 163	High TMAO group N = 91	Low TMAO group N = 72	*P* value
Age, years	36.3 (26.8, 52.8)	38.7 (29.0, 55.9)	33.6 (25.1, 50.1)	0.205
Female sex, n (%)	104 (63.8)	52 (57.1)	52 (72.2)	0.051
BMI, kg/m^2^	22.1 ± 3.9	23.0 ± 3.9	20.9 ± 3.7	0.001
6 MWD, m	409.0 (333.0, 480.0)	403.0 (267.0, 480.0)	412.0 (363.8.0, 483.8)	0.034
WHO-FC, n (%)				
I–II	112 (68.7)	54 (59.3)	58 (80.6)	0.004
III–IV	51 (31.3)	37 (40.7)	14 (19.4)	0.004
Laboratories				
TMAO, umol/L	2.0 (1.0, 3.9)	3.7 (2.4, 5.6)	0.8 (0.4, 1.3)	< 0.001
NT-proBNP, pg/ml	406.0 (139.9, 1061.0)	590.7 (204.8, 1748.5)	288.2 (98.6, 567.3)	< 0.001
Albumin, g	43.8 (40.8, 45.9)	43.2 (40.9, 46.0)	44.0 (40.7, 45.8)	0.971
Creatinine, umol/L	75.0 (65.2, 86.0)	78.0 (67.0, 91.0)	73.1 (63.5, 82.0)	0.054
Total cholesterol, mmol/L	4.1 (3.6, 5.1)	4.1 (3.5, 5.1)	4.2 (3.6, 4.9)	0.683
Echocardiography				
LVEF, %	65.0 (60.0, 70.0)	65.0 (60.3, 70.0)	65.0 (60.0, 70.0)	0.967
RVD, mm	32.0 (26.0, 37.0)	33.5 (26.0, 38.0)	30.0 (26.0, 37.0)	0.009
TAPSE, mm	16.5 ± 4.1	15.6 ± 4.3	17.7 ± 3.4	0.001
Hemodynamics				
mRAP, mmHg	6.2 ± 3.6	6.3 ± 3.7	6.0 ± 3.3	0.614
Cardiac index, L/min * m^2^	3.0 ± 1.1	2.8 ± 1.2	3.3 ± 0.9	0.005
PAWP, mmHg	9.0 (6.0, 11.0)	9.0 (6.3, 12.0)	8.0 (6.0, 10.0)	0.198
PVR, WU	8.9 ± 6.2	10.2 ± 5.5	7.4 ± 6.8	0.013
Risk stratification (COMPERA)				0.001
Low risk	73 (44.8)	32 (35.2)	41 (56.9)	
Intermediate risk	79 (48.5)	49 (53.8)	30 (41.7)	
High risk	11 (6.7)	10 (11.0)	1 (1.4)	

Variables	Total PAH patients N = 112	High TMAO group N = 58	Low TMAO group N = 54	*P* value
Treatment, n (%)				
ERAs	86 (76.8)	43 (74.1)	43 (79.6)	0.512
Prostacyclins	30 (26.8)	19 (32.8)	11 (20.4)	0.200
NO pathway	98 (87.5)	48 (82.8)	50 (92.6)	0.155
Monotherapy	20 (17.9)	12 (20.7)	8 (14.8)	0.467
Combination therapy	92 (82.1)	46 (79.3)	46 (85.2)	0.467

Variables	Total CTEPH patients N = 51	High TMAO group N = 33	Low TMAO group N = 18	*P* value
Riociguat	18 (35.3)	10 (30.3)	8 (44.4)	0.367
BPA/PEA	44 (86.3)	28 (84.8)	16 (88.9)	0.692

In this cohort, 112 PAH patients and 51 CTEPH patients were enrolled. Patients were divided into two groups according to the plasma TMAO levels (the cut-off value was 1.69 umol/L). The Kolmogorov–Smirnov was used for normality distribution test. Continuous variables were presented as mean ± standard deviation or median and interquartile ranges based on different data distributions

Categorical variables were presented as frequencies with percentages. Student’s *t*-test was used for continuous data with normal distribution, including BMI, TAPSE, mRAP, and CI, while the Wilcoxon rank-sum test was used for continuous data with non-normal distribution. Chi-square test was used for categorical variables

*TMAO* trimethylamine-N-oxide; *BMI* body mass index; *6 MWD* 6-min walk distance; *WHO-FC* world health organization function class; *NT-proBNP* N-terminal pro-brain natriuretic peptide; *LVEF* left ventricular ejection fraction; *RVD* right ventricular diameter; *TAPSE* tricuspid annular plane systolic excursion; *mRAP* mean right atrial pressure; *PAWP* pulmonary arterial wedge pressure; *PVR* pulmonary vascular resistance; *PAH* pulmonary arterial hypertension; *CTEPH* chronic thromboembolic pulmonary hypertension; *ERAs* endothelin receptor agonists; *NO* nitric oxide; *BPA* balloon pulmonary angioplasty; *PEA* pulmonary endarterectomy

Moreover, Additional file 4: Table S1 shows the clinical characteristics, including TMAO levels among IPAH, CTDPAH, and CTEPH patients. In the high-TMAO group, there were 24 patients with IPAH/HPAH, 34 patients with CHD-PAH, and 33 patients with CTEPH, while the low-TMAO group included 12, 42, and 18 patients with IPAH/HPAPH, CDH-PAH, and CTEPH, respectively. Mean TMAO levels in the IPAH/HPAH, CHD-PAH, and CTEPH cohorts were 3.1 (1.1, 4.9) μmol/L, 1.5 (0.8, 2.8) μmol/L, and 2.3 (1.2, 4.4) μmol/L, respectively.

### Association between TMAO levels and disease severity in PH patients

The correlations between TMAO levels and clinical indicators are shown in Additional file 5: Table S2. According to the results in Additional file 5: Table S2 and clinical significance, we adjusted for the confounders, including sex, body mass index, right ventricular diameter, creatinine, and hypertension, to demonstrate the real association between TMAO levels and disease severity assessed by WHO-FC, NT-proBNP, TAPSE, mixed venous oxygen saturation, risk stratification, CI, and PVR. Results showed that high TMAO levels were still associated with poor risk-associated parameters even after adjusting for confounding factors (Table 2).

**Table 2:** The associations between TMAO and disease severity in pulmonary hypertension

Logistics analysis	OR	95% CI	*P*
Model 1: WHO-FC			
Unadjusted	2.839	1.384**–**5.820	0.004
Adjusted	2.472	1.076**–**5.678	0.033
Model 2: NT-proBNP			
Unadjusted	2.659	1.398**–**5.057	0.003
Adjusted	2.572	1.184**–**5.588	0.017
Model 3: TAPSE			
Unadjusted	3.517	1.832**–**6.754	< 0.001
Adjusted	3.180	1.448**–**6.981	0.004
Model 4: Mixed venous oxygen saturation			
Unadjusted	3.257	1.264**–**8.390	0.014
Adjusted	3.806	1.050**–**13.789	0.042
Model 5: Risk stratification (low risk vs. non-low risk)			
Unadjusted	2.439	1.293**–**4.600	0.006
Adjusted	2.564	1.229**–**5.351	0.012

Linear regression analysis	β	95% CI	*P*
Model 6: Cardiac index			
Unadjusted	− 0.217	− 0.802 to − 0.141	0.005
Adjusted	− 0.257	− 0.879 to − 0.177	0.003
Model 7: PVR			
Unadjusted	0.222	0.654**–**4.908	0.011
Adjusted	0.359	1.985**–**6.477	< 0.001

Adjusted for sex, BMI, RVD, creatinine, and hypertension

TMAO was put into the models as a categorical variable with boundary of 1.69 umol/L

NT-proBNP, TAPSE, and mixed venous oxygen saturation were converted into categorical variables with boundaries of 300 pg/mL, 18 mm, and 65%, respectively

Logistics or linear regression analysis was used for analysis

*TMAO* trimethylamine-N-oxide; *CI* confidence interval; *WHO-FC* world health organization function class; *NT-proBNP* N-terminal pro-brain natriuretic peptide; *TAPSE* tricuspid annular plane systolic excursion; *PVR* pulmonary vascular resistance; *BMI* body mass index; *RVD* right ventricular diameter

### Effect of PH treatment on TMAO levels

PH was managed based on the recommendations of the European Society of Cardiology PH guidelines and important proposals from the 6th World Symposium on Pulmonary Hypertension (WSPH) [1, 11]. The treatment strategy of PAH patients in our center were shown in Additional file 2: Fig. S2. The characteristics of patients after treatment are shown in Additional file 6: Table S3. COMPERA risk stratification method, based on WHO-FC, 6MWD, and NT-proBNP, is considered a simple and effective disease assessment tool for PH patients, including PAH and CTEPH [12, 13]. In this study, patients were stratified based on COMPERA risk method before and after treatment for further analyses.

At baseline, TMAO levels differed among low (n = 73), intermediate (n = 79), and high risk (n = 11) groups (Additional file 3: Fig. S3A). After treatment, the risk stratification in 154 PH patients remained stable or improved, while it deteriorated in 9 patients. When all patients with PH were considered, post-treatment plasma TMAO levels decreased [ΔTMAO = − 0.2 (− 1.5, 0.8) μmol/L, *P* = 0.013], and a similar finding was observed in the non-deterioration population [ΔTMAO = − 0.2 (− 1.6, 0.7) μmol/L, *P* = 0.006]. Conversely, a potential increase trend in TMAO levels was observed in those with worsened risk status [ΔTMAO = 0.7 (− 0.9, 2.5) μmol/L, *P* = 0.234, Additional file 3: Fig. S3B, C].

### High TMAO levels were related to poor prognosis in patients with PH

**Fig. 2:**
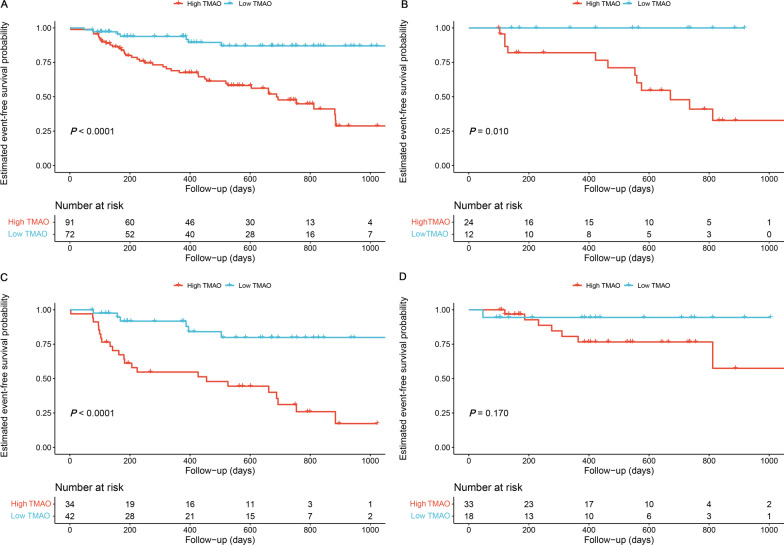
Kaplan–Meier analysis for the incidence of composite outcome events. PH patients (n = 163, *P* < 0.001 (**A**), IPAH/HPAH patients (n = 36, *P* = 0.010 (**B**), CHD-PAH (n = 76, *P* < 0.001, (**C**), and CTEPH (n = 51, *P* = 0.170 (**D**) were analysed. Composite outcome events include death, rehospitalisation due to heart failure, and at least 15% decreased 6MWD from the baseline. *P*-value calculated by the log-rank test. *TMAO* trimethylamine-N-oxide; *PH* pulmonary hypertension; *IPAH/HPAH* idiopathic/heritable pulmonary arterial hypertension; *CHD-PAH* pulmonary arterial hypertension associated with congenital heart disease; *CTEPH* chronic thromboembolic pulmonary hypertension; *6MWD* 6-min walk distance

Kaplan–Meier analysis (Fig. 2) indicated that elevated TMAO levels were associated with poor prognosis among all patients with PH (*P* < 0.001), among patients with IPAH/HPAH (*P* = 0.010), and among patients with CHD-PAH (*P* < 0.001). However, there was no significant statistical difference in the CTEPH subgroup (*P* = 0.170). The results of the univariate Cox regression analysis are shown in Additional file 7: Table S4. After adjusting for confounders, including risk stratification, treatment, and PH subtypes, both categorical and continuous TMAO levels were independent predictors for poor prognosis in patients with PH (Model 1: hazard ratio [HR] = 5.414, 95% CI 2.305–12.719; *P* < 0.001; Model 2: HR = 1.126, 95% CI 1.028–1.234; *P* = 0.011, Table 3).

**Table 3:** Multivariate Cox analysis of TMAO and prognosis in patients with PH

Variable	HR	95% CI	*P value*
Model 1			
TMAO (categorical variable)	5.414	2.305–12.719	< 0.001
WHO-FC	1.246	0.529–2.935	0.615
RVD	1.016	0.970–1.064	0.503
TAPSE	0.987	0.907–1.075	0.764
Risk stratification	1.012	0.537–1.907	0.970
Prostacyclins	1.182	0.525–2.661	0.687
BPA/PEA	0.142	0.048–0.419	< 0.001
PH subtypes	1.954	1.110–3.439	0.020
Model 2			
TMAO (continuous variable)	1.126	1.028–1.234	0.011
WHO–FC	1.758	0.764–4.046	0.185
RVD	1.019	0.972–1.067	0.435
TAPSE	0.967	0.887–1.056	0.456
Risk stratification	0.925	0.502–1.704	0.803
Prostacyclins	1.166	0.526–2.582	0.705
BPA/PEA	0.120	0.038–0.381	< 0.001
PH subtypes	2.528	1.362–4.692	0.003

*TMAO* trimethylamine-N-oxide; *WHO-FC* world health organization function class; *RVD* right ventricular diameter; *TAPSE* tricuspid annular plane systolic excursion; *BPA* balloon pulmonary angioplasty; *PEA* pulmonary endarterectomy; *PH* pulmonary hypertension

## Discussion

In this clinical study, we demonstrated that high circulating levels of the gut-microbiota-derived metabolite, TMAO, were associated with severe disease conditions and poor prognosis in patients with PH. This result highlights the strong clinical value of TMAO as a novel plasma biomarker and a potential surrogate in PH.

Even after adjusting for confounders, clinical parameters, including exercise capacity, cardiac function, haemodynamic status, and risk stratification in patients with high plasma TMAO levels, were worse than those with low levels. Moreover, Kaplan–Meier analysis in the total PH cohort and all three subgroups revealed an inverse correlation between plasma TMAO levels and PH prognosis. However, it did not reach statistical significance in the CTEPH subgroup partially due to the intersection of lines in Kaplan–Meier analysis on approximately day 200; The relatively small population size in the CTEPH subgroup may also account for this modest statistical power. More importantly, intervention with BPA in most of the CTEPH inpatients we enrolled greatly improved the outcomes and may thus blunt the effect of TMAO levels, resulting in no significant prognostic difference between high- and low-TMAO groups. Based on the result and analysis, it seems that TMAO still carries some value in predicting the outcomes of CTEPH patients; confirmation by a more comprehensive exploration, including the investigation of the changes in TMAO levels before and after BPA in a larger CTEPH cohort, is under exploration by our team.

Interestingly, the changes in plasma TMAO levels in response to PH treatment were demonstrated. We found a decrease in the total cohort, especially in patients with undeteriorated risk statusafter clinical management. The results indicated the potential value of TMAO as a surrogate marker in PH, which may contribute to better prediction and management of the disease.

In accordance with our findings, previous studies have highlighted the potential value of TMAO as a broad clinical biomarker that can aid in preventing numerous cardiovascular diseases and predicting patient prognosis and as a tool for investigating their pathogenesis. Plasma TMAO exhibits a dose-dependent association with risk stratification in patients with acute coronary syndromes [14] and increases with WHO-FC in patients with chronic heart failure [15]. Increased circulating plasma TMAO concentration increases the risk of heart failure, hypertension, and atrial fibrillation [16–18]. Although the underlying mechanism is not fully understood, previous studies have demonstrated that plasma TMAO directly contributes to the development of atherosclerosis by accelerating foam cell formation, increasing thrombotic tendency, promoting leukocyte adhesion, enhancing oxidative stress, and prompting the release of inflammatory cytokines [19–21]. Elevated plasma levels of TMAO also promote cardiac hypertrophy [22] and exacerbate cardiac dysfunction after myocardial infarction by inducing fibroblast-myofibroblast differentiation, resulting in cardiac remodelling and fibrosis [23]. However, the knowledge of TMAO in PH has been limited until now.

We speculate that the potential inner relationships between TMAO and PH are as follows. On the one hand, the elevation of TMAO may result from the abnormal haemodynamics caused by PH, and patients with high TMAO may be more likely to experience severe disease conditions. TMAO is a gut-microbiota-dependent metabolite, and any interference with the metabolic processing of TMAO may affect plasma levels [2]. One hypothesis states that increases in TMAO concentration represent a beneficial compensatory response to increased hydrostatic pressure in patients with cardiovascular diseases [24]. From this point of view, TMAO may assume a role similar to BNP, increasing with disease severity to maintain circulatory homeostasis and returning to normal after successful treatment.

Thus, improving PH status and mitigating systemic venous congestion and pathologically increased hydrostatic stress may decrease plasma TMAO levels, and higher plasma TMAO levels may herald worse prognosis in patients. Furthermore, the amelioration of PH may restore the abnormalities that elicit an increase in plasma TMAO, such as gut microbiota dysbiosis, gastrointestinal tract hyperpermeability, organ congestion, and reduced perfusion due to increased pulmonary circulation resistance, high pulmonary artery pressure, and right heart dysfunction [25, 26]. Moreover, improvements in renal perfusion after effective PH treatment may improve kidney function and enhance renal clearance of metabolites such as TMAO [25].

On the other hand, TMAO may serve as a trigger to facilitate the progression of PH and exacerbate disease severity. PH is a haemodynamic state characterised by increased mPAP at rest due to various aetiologies. The injury of vascular endothelium and the ensuing endothelial dysfunction has been determined to play a direct pathogenic role. Evidence has shown that TMAO exerted direct endothelial toxicity via numerous mechanisms, and it is tempting to speculate that TMAO aggravates PH by disturbing proper endothelial function. TMAO can directly induce and augment inflammation in vascular endothelial cells, leading to milieu alteration and consequently inducing endothelial hyperpermeability [21, 27, 28]. Research has also demonstrated that TMAO induces reactive oxygen species production and promotes endothelial cell senescence and vascular ageing [21, 28, 29], leading to further impairments in endothelial function.

The damage to vasodilatory regulation induced by TMAO may be another potential explanation. Nitric oxide (NO) is a crucial regulator of vascular tone in both systemic and pulmonary circulations and is a main therapeutic target in patients with PH [30]. It has been reported in human and animal models that high plasma TMAO levels induce NO deficiency [31], which may cause an increase in vasotension and ultimately lead to elevated pressure. Another important vasodilator, endothelium-dependent hyperpolarizing factor (EDHF), expressed especially in the distal pulmonary arterioles [32], and the impaired EDHF-mediated relaxation is a potential driver for the initiation of hypoxia-induced PH [33]. It has been reported that TMAO might affect EDHF function in the femoral arteries [34], implying a need for studies to explore whether it promotes PH by exerting similar effects in the pulmonary vasculature. In addition to decreased levels of vasodilators, some studies have reported that endothelin-1 (ET-1), a potent vasoconstrictor that participates in vascular remodelling and modulates vasomotor function (30), is significantly upregulated in mice fed with TMAO [35], through which a continuously high concentration of TMAO may indirectly worsen PH. Summarily, a further study determined to investigate the “cause” or “effect” role of TMAO in PH is warranted.

This study had a few limitations. First, it was a single-centre study, and the sample size was limited because of the rare disease incidence and strict exclusion criteria. Despite this, our work was conducted based on the largest PH centre in China and still contained a cohort convincing enough to demonstrate the value of TMAO in PH. Second, we failed to obtain dietary history or control the patients’ diets to avoid the impact of eating habits on TMAO levels. Nevertheless, we provided advanced and valuable data from a single-centre cohort regarding a brand-new biomarker in PH to improve patients’ prognoses.

## Conclusion

Our study demonstrated that PH patients with elevated TMAO concentrations exhibited more severe disease conditions and worse prognosis than those with low plasma TMAO levels. TMAO possesses the potential to be an effective plasma biomarker and a surrogate in PH, highlighting the need for a collaborative research effort over the next decade to improve and develop strategies for managing PH.

## Clinical perspectives

In the patients hospitalized for PH in China, we observed that gut microbiota-dependent trimethylamine N-oxide (TMAO) severed as a novel plasma biomarker and a latent surrogate in these population. We investigated the value of TMAO in PH characterizing high TMAO levels were associated with poor disease conditions and prognosis.

## Translational outlook

For the first time, this study unprecedentedly demonstrated the significant value of the TMAO on PH. Collaborative research effort is required over the next decade to further explore the values of gut metabolites aiming to improve and develop strategies for managing PH. If these findings are further confirmed, monitor TMAO levels in PH can be widely used due to the convenient operation in clinic.

## Supplementary Information

**Additional file 1: Figure S1.** Cut-off value of TMAO levels in study. TMAO: trimethylamine-N-oxide.

**Additional file 2: Figure S2.** Treatment strategy of PAH patients. After a comprehensive assessment on patients’ condition, monotherapy is considered in PAH patients with obesity and/or with comorbidities including diabetes, hypertension, coronary heart disease, atrial fibrillation, and lung diseases. The targeted drug finally prescribed in clinic also follows the willing of the patients and their families. PAH: pulmonary arterial hypertension; ERA: endothelin receptor antagonist; PDE5i: phosphodiesterase 5 inhibitor; PCA: prostacyclin analogue.

**Additional file 3: Figure S3.** TMAO levels in patients with different risk stratification and TMAO changes after clinical management. (A) Different TMAO levels in patients classified as low risk, intermediate risk and high risk group. (B-D) Changes of TMAO levels before and after treatment in total, non-deterioration, and deterioration patients. Analyses were explored using paired-samples t tests. *P < 0.05. TMAO: trimethylamine-N-oxide.

**Additional file 4: Table S1.** Baseline characteristics of PH patients with different etiologies.

**Additional file 5: Table S2.** Correlation between TMAO and clinical indicators.

**Additional file 6: Table S3.** Characteristics of patients with PH before and after treatment.

**Additional file 7: Table S4.** Univariate Cox regression analysis of variables.

## Data Availability

The datasets used and/or analysed during the current study are available from the corresponding author on reasonable request.

## Abbreviations

6MWD: 6-min walk distance
CHD-PAH: Pulmonary arterial hypertension associated with congenital heart disease
CTEPH: Chronic thromboembolic pulmonary hypertension
IPAH/HPAH: Idiopathic/heritable pulmonary arterial hypertension
mPAP: Mean pulmonary arterial pressure
NT-proBNP: N-terminal pro-brain natriuretic peptide
RVD: Right ventricular diameter
TAPSE: Tricuspid annular plane systolic excursion
CI: Cardiac index
TMAO: Trimethylamine N-oxide (TMAO)
WHO-FC: World Health Organization Functional Class
