# Supplementary figures and images for: Higher circulating Trimethylamine N-oxide levels are associated with worse severity and prognosis in pulmonary hypertension: a cohort study
Source: Respir Res. 2022 Dec 14;23:344. doi: 10.1186/s12931-022-02282-5 (PMC9749156; doi:10.1186/s12931-022-02282-5)

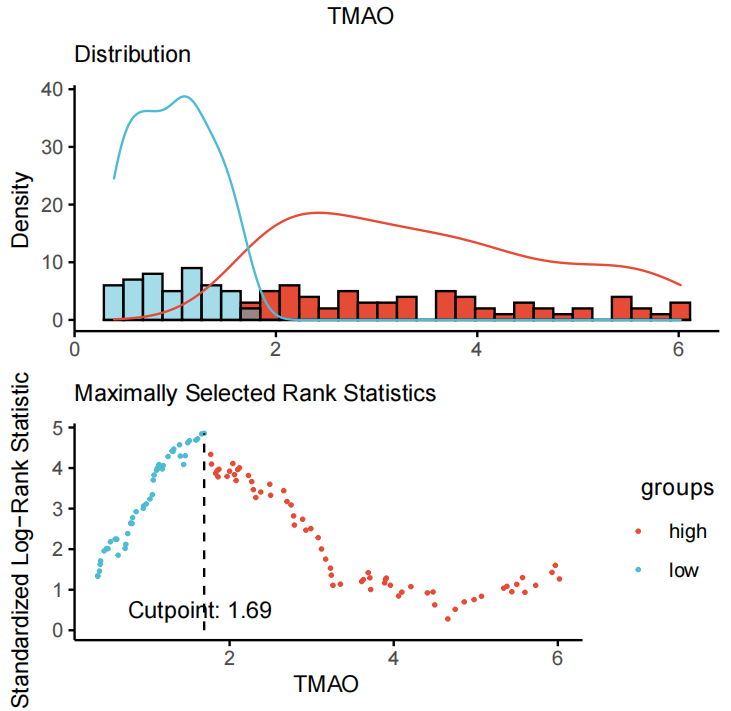


**Figure S1. Cut-off value of TMAO levels in study.** TMAO: trimethylamine-N-oxide.

Supplement: Supplementary file 1 — Additional file 1: Figure S1. Cut-off value of TMAO levels in study. TMAO: trimethylamine-N-oxide. [file 12931_2022_2282_MOESM1_ESM.docx]
