# Supplementary material for: Higher circulating Trimethylamine N-oxide levels are associated with worse severity and prognosis in pulmonary hypertension: a cohort study
Source: Respir Res. 2022 Dec 14;23:344. doi: 10.1186/s12931-022-02282-5 (PMC9749156; doi:10.1186/s12931-022-02282-5)
Supplement: Supplementary file 2 — Additional file 2: Figure S2. Treatment strategy of PAH patients. After a comprehensive assessment on patients’ condition, monotherapy is considered in PAH patients with obesity and/or with comorbidities including diabetes, hypertension, coronary heart disease, atrial fibrillation, and lung diseases. The targeted drug finally prescribed in clinic also follows the willing of the patients and their families. PAH: pulmonary arterial hypertension; ERA: endothelin receptor antagonist; PDE5i: phosphodiesterase 5 inhibitor; PCA: prostacyclin analogue. [file 12931_2022_2282_MOESM2_ESM.docx]

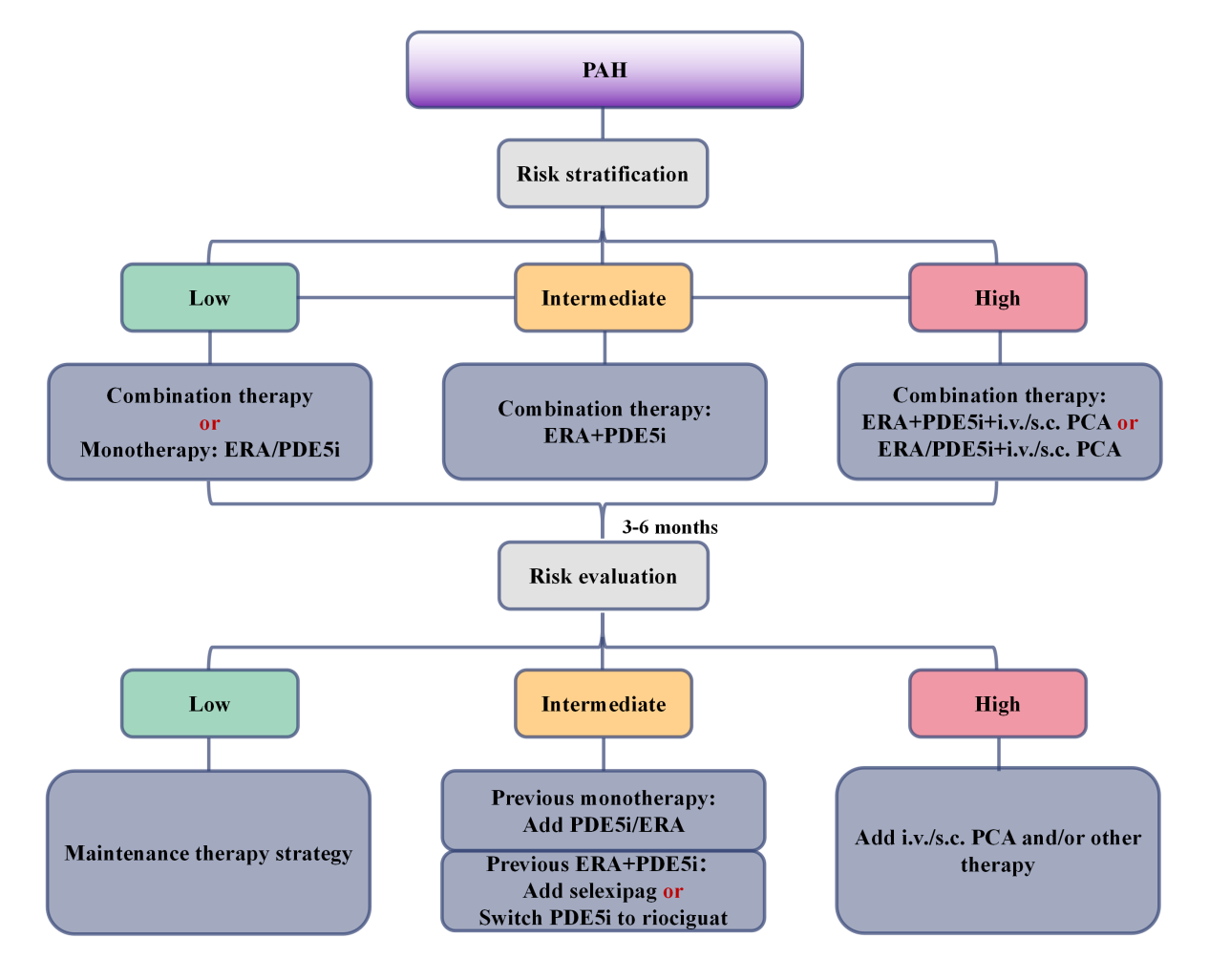


**Figure S2. Treatment strategy of PAH patients. After a comprehensive assessment on patients’ condition, monotherapy is considered in PAH patients with obesity and/or with comorbidities including diabetes, hypertension, coronary heart disease, atrial fibrillation, and lung diseases.** The targeted drug finally prescribed in clinic also follows the willing of the patients and their families. PAH: pulmonary arterial hypertension; ERA: endothelin receptor antagonist; PDE5i: phosphodiesterase 5 inhibitor; PCA: prostacyclin analogue.
