# Supplementary material for: Higher circulating Trimethylamine N-oxide levels are associated with worse severity and prognosis in pulmonary hypertension: a cohort study
Source: Respir Res. 2022 Dec 14;23:344. doi: 10.1186/s12931-022-02282-5 (PMC9749156; doi:10.1186/s12931-022-02282-5)
Supplement: Supplementary file 3 — Additional file 3: Figure S3. TMAO levels in patients with different risk stratification and TMAO changes after clinical management. (A) Different TMAO levels in patients classified as low risk, intermediate risk and high risk group. (B-D) Changes of TMAO levels before and after treatment in total, non-deterioration, and deterioration patients. Analyses were explored using paired-samples t tests. *P < 0.05. TMAO: trimethylamine-N-oxide. [file 12931_2022_2282_MOESM3_ESM.docx]

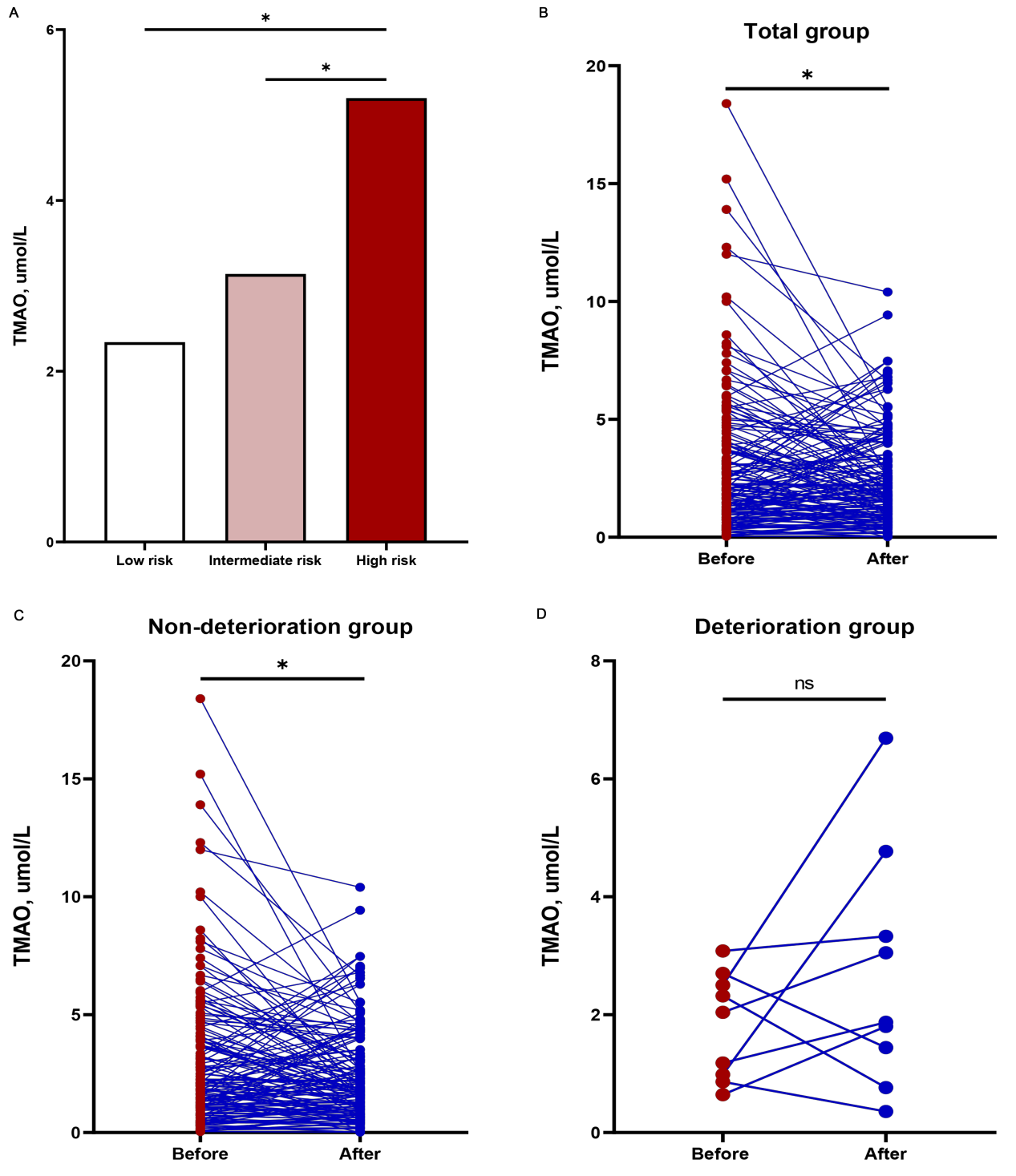


**Figure S3. TMAO levels in patients with different risk stratification and TMAO changes after clinical management.** (A) Different TMAO levels in patients classified as low risk, intermediate risk and high risk group. (B-D) Changes of TMAO levels before and after treatment in total, non-deterioration, and deterioration patients. Analyses were explored using paired-samples t tests. ****P***<0.05. TMAO: trimethylamine-N-oxide.
