# Supplementary material for: Higher circulating Trimethylamine N-oxide levels are associated with worse severity and prognosis in pulmonary hypertension: a cohort study
Source: Respir Res. 2022 Dec 14;23:344. doi: 10.1186/s12931-022-02282-5 (PMC9749156; doi:10.1186/s12931-022-02282-5)
Supplement: Supplementary file 4 — Additional file 4: Table S1. Baseline characteristics of PH patients with different etiologies. [file 12931_2022_2282_MOESM4_ESM.docx]

**Table S1. Baseline characteristics of PH patients with different etiologies**

| **Variables** | **IPAH/HPAH** | | **CHD-PAH** | | **CTEPH** | |
| --- | --- | --- | --- | --- | --- | --- |
|  | **High TMAO**  **N=24** | **Low TMAO**  **N=12** | **High TMAO**  **N=34** | **Low TMAO**  **N=42** | **High TMAO**  **N=33** | **Low TMAO**  **N=18** |
| Age, years | 34.2±10.3 | 35.9±6.8 | 33.3±12.1 | 30.8±12.4 | 55.1±15.5 | 56.9±11.2 |
| Female sex, n (%) | 16 (66.7) | 10 (83.3) | 23 (67.6) | 32 (76.2) | 13 (39.4) | 10 (55.6) |
| BMI, kg/m^2^ | 23.0±3.4 | 19.9±3.9* | 23.7±4.3 | 21.3±3.2* | 22.4±3.6 | 20.7±4.8 |
| 6 MWD, m | 375.5±131.2 | 452.6±68.6* | 372.0±142.4 | 407.7±86.3 | 379.6±123.5 | 404.9±116.3 |
| **WHO-FC, n (%)** |  |  |  |  |  |  |
| I-II | 15 (62.5) | 3 (25.0%)* | 20 (58.8) | 34 (81.0)* | 25 (75.8) | 15 (83.3) |
| III-IV | 9 (37.5) | 9 (75.0%)* | 14 (41.2) | 8 (19.0)* | 8 (24.2) | 3 (16.7) |
| **Laboratories** |  |  |  |  |  |  |
| TMAO, umol/L | 4.2 (3.1, 6.0) | 0.9 (0.5, 1.1)# | 2.9 (2.1, 4.9) | 0.8 (0.4, 1.3)# | 3.9 (2.3, 6.6) | 1.0 (0.5, 1.2)# |
| NT-proBNP, pg/ml | 1101.5 (525.8, 2057.5) | 566.3 (172.3, 1444.5)# | 544.5 (155.2, 2540.5) | 296.2 (103.2, 444.7)* | 437.5 (163.4, 1051.8) | 117.5 (61.0, 498.9)# |
| Albumin, g | 42.9±5.0 | 43.7±4.0 | 43.4±4.0 | 43.2±5.3 | 42.3±4.4 | 41.7±3.3 |
| Creatinine, umol/L | 79.0±20.5 | 73.6±16.6 | 76.6±16.1 | 72.4±14.1 | 82.8±15.9 | 82.0±17.9 |
| Total cholesterol, mmol/L | 4.3±1.2 | 4.4±0.8 | 3.8±0.8 | 4.2±1.0 | 4.8±1.0 | 4.8±1.4 |
| **Echocardiography** |  |  |  |  |  |  |
| LVEF, % | 65.5±5.3 | 65.8±3.7 | 64.0±8.6 | 65.8±6.0 | 66.7±6.5 | 61.9±15.5 |
| RVD, mm | 33.5±6.0 | 31.8±6.5 | 35.3±7.7 | 30.7±7.2* | 31.1±7.3 | 27.7±8.6 |
| TAPSE, mm | 13.7±3.7 | 17.7±3.6* | 15.5±3.8 | 17.4±3.8* | 16.9±4.7 | 18.6±2.5* |
| **Hemodynamics** |  |  |  |  |  |  |
| mRAP, mmHg | 6.2±2.4 | 4.1±3.5* | 5.8±3.1 | 6.5±2.9 | 7.1±5.1 | 6.6±3.8 |
| Cardiac index, L/min*m^2^ | 2.7±1.3 | 2.7±0.9 | 2.9±0.9 | 3.4±0.8* | 2.7±1.3 | 3.3±1.0 |
| PAWP, mmHg | 9.2±2.5 | 8.4±1.6 | 10.1±8.7 | 8.3±2.9 | 9.1±2.9 | 8.9±3.5 |
| PVR, WU | 10.3±6.0 | 12.9±8.0 | 10.1±6.0 | 7.0±6.0* | 10.2±4.6 | 4.8±6.5# |

Patients were divided into two groups according to the plasma TMAO levels (the cut-off value was 1.69 umol/L). The Kolmogorov-Smirnov or Shapiro-Wilk was used for normality distribution test. Continuous variables were presented as mean ± standard deviation or as median and interquartile range based on different data distribution. Categorical variables were presented as frequencies with percentages. Student's t-test was used for continuous data with normal distribution while Wilcoxon rank sum test was used for continuous data with non-normal distribution. Chi-square test was utilized for categorical variables. IPAH/HPAH: idiopathic/heritable pulmonary arterial hypertension; CHD-PAH: pulmonary arterial hypertension associated with congenital heart disease (CHD-PAH); CTEPH: chronic thromboembolic pulmonary hypertension; TMAO: trimethylamine-N-oxide; BMI: body mass index; 6 MWD: 6-minute walk distance; WHO-FC: world health organization function class; NT-proBNP: N-terminal pro-brain natriuretic peptide; LVEF: left ventricular ejection fraction; RVD: right ventricular diameter; TAPSE: tricuspid annular plane systolic excursion; mRAP: mean right atrial pressure; PAWP: pulmonary arterial wedge pressure; PVR: pulmonary vascular resistance. ****P*** <0.05; #***P*** <0.001.
