# Supplementary material for: Higher circulating Trimethylamine N-oxide levels are associated with worse severity and prognosis in pulmonary hypertension: a cohort study
Source: Respir Res. 2022 Dec 14;23:344. doi: 10.1186/s12931-022-02282-5 (PMC9749156; doi:10.1186/s12931-022-02282-5)
Supplement: Supplementary file 5 — Additional file 5: Table S2. Correlation between TMAO and clinical indicators. [file 12931_2022_2282_MOESM5_ESM.docx]

**Table S2. Correlation between TMAO and clinical indicators**

| **Variables** | ***r*** | ***P* value** |
| --- | --- | --- |
| Age, years | 0.100 | 0.206 |
| Sex, female | -0.156 | **0.047** |
| BMI, kg/m^2^ | 0.281 | **<0.001** |
| 6MWD, m | -0.104 | 0.207 |
| WHO-FC | 0.227 | **0.004** |
| **Laboratories** |  |  |
| NT-proBNP, pg/ml (categorical variable) | 0.236 | **0.002** |
| Albumin, g | -0.003 | 0.971 |
| Creatinine, umol/L | 0.152 | 0.054 |
| Total cholesterol, mmol/L | -0.032 | 0.685 |
| **Echocardiography** |  |  |
| LVEF, % | -0.003 | 0.967 |
| RVD, mm | 0.206 | **0.009** |
| TAPSE, mm (categorical variable) | -0.304 | **<0.001** |
| **Hemodynamics** |  |  |
| mRAP, mmHg | -0.001 | 0.992 |
| Cardiac index, L/min*m^2^ | -0.220 | **0.005** |
| PAWP, mmHg | 0.131 | 0.199 |
| PVR, WU | 0.311 | **<0.001** |

TMAO was converted into a categorical variable with boundary of 1.69 umol/L. Spearman’s correlation (two-tailed) analyses were used for exploring the correlations between TMAO with clinical variables.

TMAO: trimethylamine-N-oxide; BMI: body mass index; 6 MWD: 6-minute walk distance; WHO-FC: world health organization function class; NT-proBNP: N-terminal pro-brain natriuretic peptide; LVEF: left ventricular ejection fraction; RVD: right ventricular diameter; TAPSE: tricuspid annular plane systolic excursion; mRAP: mean right atrial pressure; PAWP: pulmonary arterial wedge pressure; PVR: pulmonary vascular resistance.
