# Supplementary material for: Higher circulating Trimethylamine N-oxide levels are associated with worse severity and prognosis in pulmonary hypertension: a cohort study
Source: Respir Res. 2022 Dec 14;23:344. doi: 10.1186/s12931-022-02282-5 (PMC9749156; doi:10.1186/s12931-022-02282-5)
Supplement: Supplementary file 6 — Additional file 6: Table S3. Characteristics of patients with PH before and after treatment. [file 12931_2022_2282_MOESM6_ESM.docx]

**Table S3. Characteristics of patients with PH before and after treatment**

| **Variables** | **PH patients at baseline** | **PH patients after treatment** |
| --- | --- | --- |
| BMI, kg/m^2^ | 22.1±3.9 | 22.0±3.6 |
| 6 MWD, m | 409.0 (333.0, 480.0) | 447.0 (397.5, 490)* |
| **WHO-FC, n (%)** |  |  |
| I-II | 112 (68.7) | 133 (81.6)* |
| III-IV | 51 (31.3) | 30 (18.4)* |
| **Laboratories** |  |  |
| TMAO, umol/L | 2.0 (1.0, 3.9) | 1.8 (0.9, 3.3)* |
| NT-proBNP, pg/ml | 406.0 (139.9, 1061.0) | 238.3 (85.3, 733.6)* |
| Albumin, g | 43.8 (40.8, 45.9) | 44.0 (40.9, 46.2) |
| Creatinine, umol/L | 75.0 (65.2, 86.0) | 74.4 (66.0, 86.0) |
| Total cholesterol, mmol/L | 4.1 (3.6, 5.1) | 4.4 (3.7, 4.9) |
| **Echocardiography** |  |  |
| LVEF, % | 65.0 (60.0, 70.0) | 65.0 (63.0, 70.0) |
| RVD, mm | 32.0 (26.0, 37.0) | 30.0 (26.0, 37.0)* |
| TAPSE, mm | 16.5±4.1 | 17.6±3.6* |
| **Hemodynamics** |  |  |
| mRAP, mmHg | 6.2±3.6 | 6.0±3.5 |
| Cardiac index, L/min*m^2^ | 3.0±1.1 | 3.3±0.8* |
| PAWP, mmHg | 9.0 (6.0, 11.0) | 9.0 (6.5, 12.0) |
| PVR, WU | 8.9±6.2 | 7.7±5.3* |
| **Risk stratification (COMPERA)** |  |  |
| Low risk | 73 (44.8) | 104 (63.8)* |
| Intermediate risk | 79 (48.5) | 50 (30.7)* |
| High risk | 11 (6.7) | 9 (5.5) |

The Kolmogorov-Smirnov was used for normality distribution test. Continuous variables were presented as mean ± standard deviation or as median and interquartile range based on different data distribution. Categorical variables were presented as frequencies with percentages. Student's t-test was used for continuous data with normal distribution while Wilcoxon rank sum test was used for continuous data with non-normal distribution. Chi-square test was utilized for categorical variables. TMAO: trimethylamine-N-oxide; BMI: body mass index; 6 MWD: 6-minute walking distance; WHO-FC: world health organization function class; NT-proBNP: N-terminal pro-brain natriuretic peptide; LVEF: left ventricular ejection fraction; RVD: right ventricular diameter; TAPSE: tricuspid annular plane systolic excursion; mRAP: mean right atrial pressure; PAWP: pulmonary arterial wedge pressure; PVR: pulmonary vascular resistance; * ***P*** <0.05.
