# Supplementary material for: Higher circulating Trimethylamine N-oxide levels are associated with worse severity and prognosis in pulmonary hypertension: a cohort study
Source: Respir Res. 2022 Dec 14;23:344. doi: 10.1186/s12931-022-02282-5 (PMC9749156; doi:10.1186/s12931-022-02282-5)
Supplement: Supplementary file 7 — Additional file 7: Table S4. Univariate Cox regression analysis of variables. [file 12931_2022_2282_MOESM7_ESM.docx]

**Table S4. Univariate Cox regression analysis of variables**

| **Variable** | **HR** | **95% CI** | ***P*** |
| --- | --- | --- | --- |
| Age, year | 0.998 | 0.970**–**1.007 | 0.225 |
| Sex, female | 0.617 | 0.353**–**1.076 | 0.089 |
| BMI, kg/m^2^ | 1.068 | 0.992**–**1.149 | 0.082 |
| 6 MWD | 0.998 | 0.996**–**1.001 | 0.212 |
| WHO-FC | 2.186 | 1.244**–**3.844 | **0.007** |
| TMAO, umol/L (categorical variable) | 5.648 | 2.541**–**12.555 | **<0.001** |
| TMAO, umol/L (continuous variable) | 1.114 | 1.039**–**1.194 | **0.002** |
| NT-proBNP, pg/mL (categorical variable) | 1.649 | 0.900**–**3.020 | 0.106 |
| Albumin, g | 0.928 | 0.872**–**0.988 | **0.019** |
| Creatinine, umol/L | 0.994 | 0.979**–**1.010 | 0.495 |
| Total cholesterol, mmol/L | 0.612 | 0.451**–**0.831 | **0.002** |
| LVEF, % | 0.999 | 0.966**–**1.033 | 0.963 |
| RVD, mm | 1.055 | 1.013**–**1.100 | **0.011** |
| TAPSE, mm | 0.906 | 0.841**–**0.975 | **0.009** |
| mRAP, mmHg | 0.981 | 0.891**–**1.080 | 0.698 |
| CI, L/(min*m^2^) | 0.907 | 0.710**–**1.159 | 0.436 |
| PAWP, mmHg | 0.986 | 0.883**–**1.101 | 0.807 |
| PVR, WU | 0.998 | 0.950**–**1.049 | 0.953 |
| Risk stratification | 1.826 | 1.183**–**2.820 | **0.007** |
| ERAs | 0.909 | 0.524**–**1.576 | 0.734 |
| Prostacyclins | 2.166 | 1.164**–**4.030 | 0.015 |
| NO pathway drug | 1.141 | 0.596**–**2.184 | 0.691 |
| Monotherapy | 1.298 | 0.724**–**2.326 | 0.381 |
| Dual therapy | 0.765 | 0.438**–**1.336 | 0.347 |
| Triple therapy | 2.493 | 1.053**–**5.903 | **0.038** |
| BPA/PEA | 0.360 | 0.154**–**0.845 | **0.019** |
| Disease type |  |  | 0.111 |
| IPAH (HPAH) /CTEPH | 1.353 | 0.582**–**3.142 | 0.482 |
| CHD-PAH/CTEPH | 2.063 | 1.005**–**4.234 | **0.049** |

TMAO: trimethylamine-N-oxide; CI: confidence interval; BMI: body mass index; 6 MWD: 6-minute walk distance; WHO-FC: world health organization function class; NT-proBNP: N-terminal pro-brain natriuretic peptide; LVEF: left ventricular ejection fraction; RVD: right ventricular diameter; TAPSE: tricuspid annular plane systolic excursion; mRAP: mean right atrial pressure; PAWP: pulmonary arterial wedge pressure; PVR: pulmonary vascular resistance; ERAs: endothelin receptor agonists; NO: nitric oxide; BPA: balloon pulmonary angioplasty; PEA: pulmonary endarterectomy; IPAH/HPAH: idiopathic/heritable pulmonary arterial hypertension; CHD-PAH: pulmonary arterial hypertension associated with congenital heart disease (CHD-PAH); CTEPH: chronic thromboembolic pulmonary hypertension.
